# Mitophagy in idiopathic pulmonary fibrosis: from molecular mechanisms to therapeutic opportunities

**DOI:** 10.3389/fcell.2026.1925197

**Published:** 2026-09-07

**Authors:** Mengzhu He, Yi Li, Hong Huang, Zhaotong Cong, Yongfeng Yang

**Affiliations:** 1 Department of Pulmonary and Critical Care Medicine, Institute of Respiratory Health and Multimorbidity, Institute of Respiratory Health, State Key Laboratory of Respiratory Health and Multimorbidity, Frontiers Science Center for Disease-related Molecular Network, Sichuan Provincial Engineering Laboratory of Precision Medicine, Precision Medicine Key Laboratory of Sichuan Province, West China Hospital, West China School of Medicine, Sichuan University, Chengdu, Sichuan, China; 2 Tianfu Jincheng Laboratory, Chengdu, China; 3 Institute of Herbgenomics, Chengdu University of Traditional Chinese Medicine, Chengdu, Sichuan, China

**Keywords:** biomarkers, cellular senescence, combination therapy, idiopathic pulmonary fibrosis, mitophagy, Pink1/parkin

## Abstract

Idiopathic pulmonary fibrosis (IPF) is a progressive, fatal interstitial lung disease with limited therapeutic options and a median survival of only 3–5 years. Although antifibrotic agents such as pirfenidone (PFD) and nintedanib can decelerate functional decline, they fail to reverse established fibrosis, underscoring an urgent need for novel therapeutic paradigms. Emerging evidence has positioned mitophagy—the selective autophagic clearance of damaged mitochondria—at the nexus of IPF pathogenesis. In this review, we systematically dissect the regulatory networks governing mitophagy, encompassing the canonical PINK1/Parkin pathway, receptor-mediated mechanisms (BNIP3/NIX/FUNDC1), and their intricate cross-talk with endoplasmic reticulum stress (ERS) and ferroptosis, highlighting how these interconnected pathways converge to determine alveolar epithelial cell (AEC) fate, fibroblast activation, and inflammatory reprogramming. Notably, the pathological impact of mitophagy is highly cell-type-specific and context-dependent, exhibiting protective functions in epithelial cells while paradoxically promoting pro-fibrotic phenotypes in macrophages under certain conditions, which poses both challenges and opportunities for therapeutic intervention. Furthermore, we critically evaluate emerging pharmacological and biological strategies targeting mitophagy, and propose that future combination regimens—guided by non-invasive mitophagy biomarkers—may overcome current clinical bottlenecks. By integrating mechanistic insights with translational perspectives, this review provides a roadmap for developing mitophagy-targeted interventions as a next-generation therapeutic paradigm for IPF.

## 1 Introduction

IPF is a chronic, progressive fibrosing interstitial pneumonia of unknown etiology. Affecting approximately three million people worldwide, IPF has a median survival of only 3–5 years from the time of diagnosis—a prognosis worse than that of many malignancies (Richeldi et al., 2017). Despite recent therapeutic advances, the two FDA-approved antifibrotic agents, PFD and nintedanib, only slow disease progression without reversing established fibrosis, and are limited by side effects and variable efficacy (Spagnolo et al., 2021). Lung transplantation, while potentially curative, remains available to only a minority of patients due to donor scarcity and surgical contraindications. Although mitophagy is also implicated in lung cancer pathogenesis, this topic is beyond the scope of the current review. Thus, there is an urgent need to identify novel pathogenic mechanisms and therapeutic targets.

Mitochondria have long been recognized as the cellular powerhouses; however, they are now increasingly appreciated as central integrators of cell health, stress responses, and programmed cell death (Shaghaghi et al., 2021; Ziegler et al., 2015; Zemirli et al., 2018). In IPF, accumulating evidence implicates mitochondrial dysfunction—manifested by excessive reactive oxygen species (ROS) production, impaired energy metabolism, and mitochondrial DNA (mtDNA) release—as a critical driver of disease initiation and progression (Shaghaghi et al., 2021; Bueno et al., 2019). Mitophagy, the selective autophagic elimination of damaged mitochondria, serves as a key quality control mechanism that preserves mitochondrial homeostasis and determines cell fate (Yamashita and Kanki, 2017). While the core molecular machinery of mitophagy—including the PINK1/Parkin pathway and receptor-mediated routes involving BNIP3, NIX, and FUNDC1—has been well characterized, emerging data reveal intricate cross-regulatory networks connecting mitophagy with ERS and ferroptosis, adding new layers of complexity to our understanding of IPF pathogenesis.

The pathophysiology of IPF involves a triad of interconnected processes: persistent AEC injury, aberrant fibroblast activation and myofibroblast differentiation, and dysregulated extracellular matrix (ECM) remodeling (Heukels et al., 2019). Notably, mitophagy appears to play a dual, context-dependent role within this triad—it exerts protective effects in AECs by limiting apoptosis and senescence, yet may paradoxically promote pro-fibrotic phenotypes in macrophages under certain conditions. This cell-type-specific and stage-dependent heterogeneity poses both challenges and opportunities for therapeutic intervention. Several preclinical studies have demonstrated that pharmacological or genetic modulation of mitophagy can attenuate fibrosis, and early clinical data suggest that agents such as metformin or mesenchymal stem cell (MSC)-based therapies may confer benefit through mitophagy-related mechanisms (Kurita et al., 2017; Huang et al., 2023; Teague et al., 2022).

Although the relationship between mitophagy and IPF has been addressed in previous reviews, a comprehensive synthesis integrating the latest mechanistic advances—particularly the interplay with ERS, ferroptosis, and emerging therapeutic strategies—is lacking. Moreover, the translational gap between preclinical findings and clinical application remains underexplored. In this review, we systematically summarize the current understanding of mitophagy regulatory networks in IPF, critically evaluate contradictory findings across cell types and disease stages, and discuss the clinical potential of mitophagy-targeted interventions, including combination regimens and biomarker-guided patient stratification. By bridging fundamental mechanisms with translational perspectives, we aim to provide a conceptual framework for developing mitophagy-centered therapeutic paradigms in IPF.

## 2 Mitochondrial autophagy and its regulatory mechanism

When cells are subjected to external stimuli such as reactive oxygen species (ROS), nutritional deficiencies, and cellular senescence, mitochondria may depolarize and lose their membrane potential, which triggers a selective autophagic mechanism that degrades the mitochondria, called mitochondrial autophagy (Ashrafi and Schwarz, 2013; Johansen and Lamark, 2020). Mitochondrial autophagy is a special autophagic process that accurately recognizes and removes damaged mitochondria from the cell as a means of maintaining the stability of the cell’s internal environment, in which the autophagosome wraps around the damaged mitochondria and fuses with the lysosome, and the hydrolytic enzymes in the lysosome break down the mitochondria and digest them, completing the removal process state (Goodall et al., 2022). Since the concept of mitochondrial autophagy was first introduced in 2005 (Lemasters, 2005), mitochondrial autophagy has become a popular research topic because of its increasing role in a variety of human pathologies and its association with a wide range of diseases, including neurodegeneration, cardiovascular and respiratory disorders, and metabolic diseases (Palikaras et al., 2018; Xu et al., 2020; Dikic and Elazar, 2018; Harrington et al., 2023; Onishi et al., 2021). Mitochondrial autophagy has an important role in the development of lung diseases, and its functional abnormalities are closely related to the pathogenesis of a variety of lung diseases, which are more prominent in chronic obstructive pulmonary disease (COPD) and IPF (Table 1).

**TABLE 1:** Summary of literature on the involvement of mitochondrial autophagy in the development of COPD.

Performance	Experimental model	Mitochondrial autophagy-related targets	Functionality	References
Mitophagy promotes COPD	COPD patients	BNIP3L, FUNDC1 and Parkin	↑BNIP3L/FUNDC1and↑Parkin→↑mitophagy→muscle mitochondrial loss	(Leermakers et al., 2018)
	Human bronchial epithelial cells (Beas-2B)	DRP1 and PINK1	Roflumilast inhibits CSE-induced ↑p-DRP1/PINK1 → ↓mitophagy → ↓cell death	(Kyung et al., 2018)
	Human bronchial epithelial cells (Beas-2B and NHBE)		Quercetin suppresses mitophagy-dependent apoptosis via ↓caspase activity	(Son et al., 2018)
	Bronchial epithelial cells (BEAS-2B)	FUNDC1	USP19 silencing → ↓FUNDC1 → ↓mitophagy → alleviates COPD	(You et al., 2023; Wen et al., 2019)
	Human bronchial epithelial cells (Beas-2B) and mouse model of COPD	FUNDC1-DRP1	FUNDC1 silencing blocks mitophagy and epithelial apoptosis via DRP1 interaction	(Wen et al., 2019)
	Mouse and bronchial epithelial cells (BEAS-2B)	PINK1, Parkin and DRP1	PM2.5 → ↑NOX4/Nrf2 redox imbalance → ↑ROS → ↑mitophagy → AECOPD	(Fan et al., 2023)
	Human bronchial epithelial cells (BEAS-2B) and mouse	LC3 II/I and PINK1	Ginsenoside Rg3 activates SIRT1 → ↓mitophagy → improves COPD	(Wang et al., 2024a)
	Human bronchial epithelial cells (BEAS-2B)	LC3II, p-Drp and PINK1	Thymoquinone ↓LC3II/p-Drp/PINK1 → ↓mitophagy and ↓necroptosis	(Dera et al., 2020; Zhang et al., 2019)
	Airway epithelial cell	Nix/BNIP3L	CSE ↑Nix → ↑mitophagy → epithelial injury	(Zhang et al., 2019)
	Lung epithelial cells and mouse models	PINK1	↑PINK1 and RIP3 → mitophagy-dependent necroptosis → COPD pathogenesis	(Mizumura et al., 2014)
	Lung bronchial epithelial cell line (BEAS-2B)		TROP2 ↑DRP1 → ↑PINK1-mediated mitophagy → accelerates age-related COPD	(Zhao and Wu, 2024)
	Rats/Rat		NEAT1/PINK1 pathway → ↑mitophagy → COPD progression	(Lin et al., 2022)
	Mouse and tracheal epithelial cells (BEAS-2B)	PINK1-Parkin	PKM2 regulates CS-induced COPD via ↑PINK1/Parkin mitophagy	(Li D. et al., 2022)
	Human bronchial epithelial cells (BEAS-2B)		STAT3 activation → ↑PINK1/Parkin mitophagy and TGF-β1 → EMT	(Wei et al., 2024)
	Human airway epithelial cell	ULK1	CS activates MAPK15-ULK1 → ↑mitophagy	(Zhang M. et al., 2021)
Mitophagy slows COPD	Human bronchial epithelial cells (BEAS-2B)	Parkin	H_2_S → ↑Klotho/IGF-1R/Parkin mitophagy → attenuates PM2.5-induced COPD	(Wang et al., 2025)
	Human/mouse lung fibroblasts (HFL1)		Restoration of mitophagy delays cellular senescence → therapeutic benefit	(Ahmad et al., 2015)
	Mouse and bronchial epithelial cells (BEAS-2B)		Parkin overexpression (not PINK1) rescues mitophagy → attenuates COPD	(Araya et al., 2019)
	Airway epithelial cell	PINK1	ECC-BYF III promotes mitophagy via NRF2/PINK1 → ameliorates senescence	(Li M. Y. et al., 2022)
	Fibroblast		HO-1 restores mitophagy → attenuates fibroblast senescence	(Even et al., 2018)
	AECs (L-132) and mouse	PINK-1, Parkin and LC3B-II	Melatonin ↑PINK1/Parkin/LC3B-II → ↑mitophagy → inhibits NLRP3 and ameliorates COPD	(Mahalanobish et al., 2020)
	Bronchial epithelial cells (HBEC) and COPD patients	PINK1-Parkin	PINK1/Parkin → ↑mitophagy → ↓ROS and ↓senescence	(ITO et al., 2015)
	Bone marrow-derived macrophages (BMDM)		↑p-MKK3 leads to insufficient mitophagy → exacerbates COPD	(Mannam et al., 2016)
	Alveolar (A549) and airway (BEAS-2B) epithelial cells	DRP1, MFF, SQSTM1/p62 and LC3B-II/I	UA ↑LC3B-II/I and ↓DRP1/MFF/p62 → ↑mitophagy → prevents epithelial injury	(Wang S. et al., 2023)
	L6 myoblasts and rat	LC3B, ULK1, PINK1 and Parkin	BJF inhibits mitophagy via AMPK → improves COPD	(Wang S. et al., 2023)

### 2.1 Overview of mitophagy pathways

**FIGURE 1:**
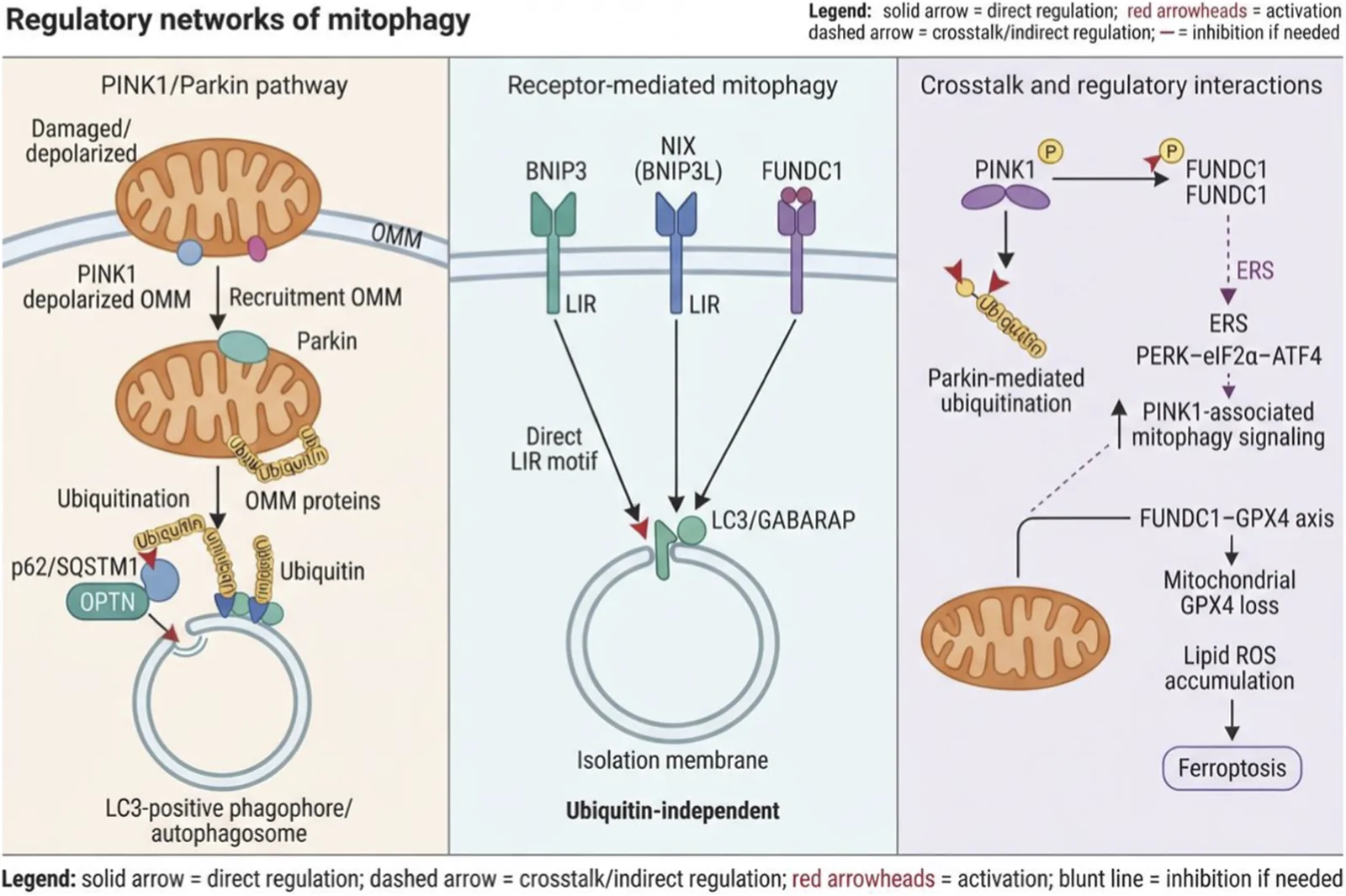
Regulatory networks of mitophagy This figure illustrates the core molecular machinery of mitophagy and its cross-talk with endoplasmic reticulum stress (ERS) and ferroptosis. Solid arrows indicate direct regulation; dashed arrows denote cross-talk/indirect regulation; red arrowheads represent activation; blue T-bars represent inhibition (Left panel): PINK1/Parkin pathway: Upon mitochondrial damage, PINK1 accumulates on the outer mitochondrial membrane (OMM), recruiting and activating Parkin. Activated Parkin ubiquitinates OMM substrates, which are recognized by autophagy receptors (p62/SQSTM1, OPTN) that simultaneously bind ubiquitin and LC3, tethering damaged mitochondria to autophagosomes for lysosomal degradation (Middle panel): Receptor-mediated mitophagy:OMM proteins BNIP3, NIX, and FUNDC1 directly bind LC3 via their LIR motifs, recruiting the autophagic machinery in a ubiquitin-independent manner (Right panel): Cross-talk and regulatory interactions: The two pathways interconnect through: (i) Parkin-mediated ubiquitination of BNIP3/NIX/FUNDC1; (ii) PINK1-mediated phosphorylation of FUNDC1 to enhance LC3 binding; (iii) ERS-induced PERK–ATF4 axis upregulating PINK1/Parkin transcription; and (iv) the FUNDC1–GPX4 axis linking mitophagy to ferroptosis via autophagic degradation of GPX4, leading to lipid ROS accumulation.

With the in-depth study of mitophagy, multiple signaling pathways have been identified, among which the PINK1/Parkin pathway and receptor-mediated mitophagy are the most extensively characterized (Egan et al., 2011). The major mitophagy pathways are schematically summarized in Figure 1.

### 2.2 The PINK1/Parkin pathway

The PINK1/Parkin pathway plays a pivotal role in the initiation and regulation of mitophagy (Madeo et al., 2015). Under physiological conditions, PTEN-induced kinase 1 (PINK1) is continuously imported into mitochondria and degraded. Upon mitochondrial damage, PINK1 import is arrested, leading to its accumulation on the outer mitochondrial membrane (Bingol and Sheng, 2016). This accumulation recruits and activates the E3 ubiquitin ligase Parkin by altering its spatial conformation, enabling it to ubiquitinate various mitochondrial outer membrane proteins (Riley et al., 2013). These ubiquitinated mitochondria are subsequently recognized by autophagic adaptors (e.g., p62/SQSTM1, OPTN, NDP52) and engulfed by autophagosomes for lysosomal degradation.

### 2.3 Receptor-mediated mitophagy

In contrast to the ubiquitin-dependent pathway, receptor-mediated mitophagy is initiated through direct interaction between mitochondrial outer membrane proteins and the autophagy protein LC3. In mammals, three main mitophagy receptors have been identified—BNIP3, NIX (also known as BNIP3L), and FUNDC1—each of which contains a canonical LC3-interacting region (LIR) motif that mediates the formation of autophagosomes around damaged mitochondria (Mizushima et al., 2008).

### 2.4 Cross-talk between PINK1/Parkin- and receptor-mediated mitophagy

The PINK1/Parkin pathway and receptor-mediated mitophagy do not operate independently; instead, they engage in complex cross-regulatory networks. Outer mitochondrial membrane receptors such as BNIP3, NIX, and FUNDC1 can not only directly recruit the autophagic machinery via their LC3-interacting region (LIR) motifs, but also serve as substrates for Parkin-mediated ubiquitination, thereby integrating the two regulatory modes (Mizushima et al., 2008; Gu et al., 2025). Moreover, PINK1 can phosphorylate FUNDC1 to enhance its binding affinity for LC3, suggesting that phosphorylation and ubiquitination act synergistically at the receptor level (Wen et al., 2019). This cross-talk allows cells to flexibly initiate mitophagy through different routes or combinations thereof, depending on the type and intensity of stress signals, ensuring robust mitochondrial quality control.

### 2.5 Interplay between mitophagy and ERS

ERS and mitophagy are closely linked in the pathogenesis of IPF. ERS can transcriptionally regulate PINK1 and Parkin expression via the PERK–ATF4 pathway (Bouman et al., 2011), while activated mitophagy eliminates dysfunctional mitochondria and reduces mitochondrial-derived reactive oxygen species (mtROS), thereby alleviating ERS-induced apoptosis and senescence (Lawson et al., 2011; Bueno et al., 2018). However, excessive or sustained ERS may suppress Parkin expression, leading to defective mitophagy and establishing a vicious cycle of ERS–mitochondrial dysfunction (Bueno et al., 2015). This bidirectional relationship suggests that maintaining the balance between ERS and mitophagy may be a critical intervention point in IPF.

### 2.6 Interplay between mitophagy and ferroptosis

Ferroptosis, a form of programmed cell death driven by lipid peroxidation, has recently been implicated in IPF pathogenesis. Emerging evidence shows that the mitophagy receptor FUNDC1 interacts with glutathione peroxidase 4 (GPX4), facilitating GPX4 entry into mitochondria and its subsequent autophagic degradation, thereby promoting ferroptosis (Bi et al., 2024). This mechanism offers a novel explanation for the paradoxical observation that enhanced mitophagy can exacerbate cell injury. In IPF, elevated PINK1 expression in AECs coexists with aggravated mitochondrial damage; whether this is linked to FUNDC1–GPX4-mediated ferroptosis warrants further investigation. Additionally, the interplay between ferroptosis and mitophagy in fibroblast activation remains poorly understood and may represent a new avenue for combination therapy.

## 3 The role of mitochondrial autophagy in IPF

**FIGURE 2:**
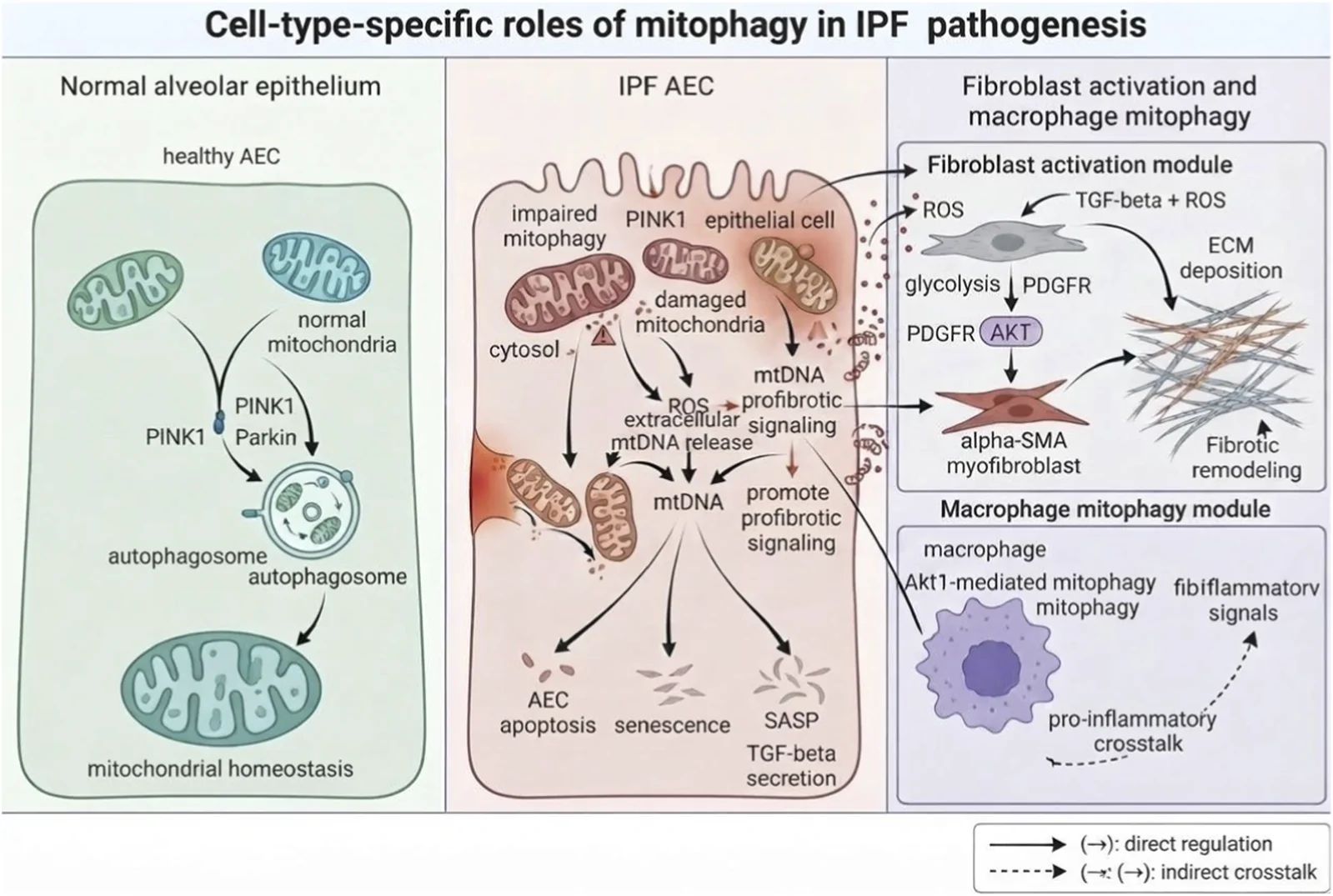
Cell-type-specific roles of mitophagy in IPF pathogenesis This figure depicts the divergent and context-dependent roles of mitophagy across alveolar epithelial cells (AECs), fibroblasts, and macrophages in IPF. Solid arrows (→) indicate direct regulation; dashed arrows (⇢) denote indirect cross-talk; red indicates pathological/promoting effects; blue indicates protective effects. Left panel (Normal AECs): Intact PINK1/Parkin-mediated mitophagy maintains mitochondrial homeostasis and epithelial integrity. Middle panel(IPF AECs): PINK1 downregulation leads to defective mitophagy, resulting in ROS/mtDNA accumulation, cGAS-STING activation, SASP induction, and secretion of pro-fibrotic factors (TGF-β, IL-6, IL-8), driving AEC apoptosis, senescence, and barrier disruption. Right upper panel (Fibroblasts): TGF-β and ROS from injured epithelia promote fibroblast activation. Defective mitophagy in fibroblasts sustains ROS production, potentiates PDGFR/PI3K/AKT and TGF-β/Smad3 signaling, and drives a glycolytic switch (HIF-1α stabilization), leading to myofibroblast differentiation (α-SMA^+^) and excessive ECM deposition. Right bottom panel (Macrophages): Mitophagy exerts dual effects in macrophages: Akt1-mediated mitophagy promotes mtROS and TGF-β1 secretion (pro-fibrotic), whereas miR-33 deficiency or OGG1 inhibition enhances mitophagy while reducing inflammation (anti-fibrotic), highlighting the need for cell-type-specific modulation. Integrated vicious cycle: Defective mitophagy in AECs drives TGF-β release → fibroblast activation → ECM production → inflammatory signals, which further reinforce epithelial injury and fibrotic progression, forming a self-perpetuating loop.

Studies have shown that the efficiency of mitochondrial autophagy is closely related to age-related diseases, and the efficiency of mitochondrial autophagy decreases with age. But IPF is a disease associated with cellular senescence, indicating that mitochondrial autophagy plays an extremely important role in the development of IPF. The critical role of mitochondrial autophagy in fibrotic lung disease has become an area of current research interes (Mora et al., 2017; Nakahira et al., 2016; Sun et al., 2016; Araya et al., 2013), which has a very important but as yet undefined role in a variety of human diseases and is associated with lung-related diseases and their progression (Geisler et al., 2010; Katzen and Beers, 2020; Zhang Y. et al., 2021), the pathogenesis of IPF is complex and involves sustained injury to AECs, aberrant fibroblast activation, and ECM remodeling imbalance and other multiple processes (Heukels et al., 2019). Table 2 summarizes the recent research progress on the role of mitochondrial autophagy in IPF. The cell-type-specific roles of mitophagy in IPF pathogenesis are depicted in Figure 2.

**TABLE 2:** Recent research progress on the role of mitochondrial autophagy in IPF.

Performance	Experimental model	Mitochondrial autophagy-related targets	Functionality	References
AEC apoptosis	Primary AECII (IPF patients/mice)	PINK1	PINK1 deficiency → defective mitophagy → ↑AECII apoptosis and fibrosis in aged lungs	(Bueno et al., 2015)
	Beas-2B cells/mouse	PINK1	TGF-β1-induced PINK1 protects against apoptosis; PINK1^−/−^ mice show ↑AECII apoptosis and fibrosis	(Patel et al., 2015)
	Lung epithelial cells (SAEC, AECII and MLE12)	PINK1	Thyroid hormone ↑PINK1 → ↓Bax and ↑Bcl-xl → restores mitochondria and ↓apoptosis	(Yu et al., 2018)
	Mouse and human alveolar epithelioid cells (A549)	PINK1	MUTYH KO → ↑PINK1 → ↑mitophagy → ↓Bax/Cleaved caspase-3 → ↓apoptosis and fibrosis	(Sun et al., 2020)
	primary AECII cells (mice)	PINK1	IL-17A ↓PINK1 → ↓mitophagy → ↑AECII apoptosis → promotes fibrosis	(Xiao et al., 2022)
	Human alveolar epithelioid cells (A549)	PINK1-Parkin	FBR-2 activates PINK1/Parkin mitophagy → blocks mitochondrial apoptosis	(Gu et al., 2022)
	Human alveolar epithelioid cells (A549)	PINK1 and LC3BII/LC3B I	PGAM5 deficiency → ↓PINK1-mediated mitophagy → ↓bleomycin cytotoxicity and ↓apoptosis	(Ganzleben et al., 2019)
	Type II AECs and mouse	BNIP3-FUNDC1	PINK1 deficiency upregulates BNIP3/FUNDC1 → compensatory mitophagy → alleviates fibrosis	(Gu et al., 2025)
AEC senescence	Human alveolar epithelioid-like cells (A549 and ATII)	LC3BII,P62 and VADC1	EF24 ↑LC3BII and ↓p62/VDAC1 → ↑mitophagy → reverses senescence and fibrosis	(Zhang et al., 2024)
	Lung epithelial cells (MLE-12)	PINK1-Parkin	Tetrandrine (TET) promotes PINK1/Parkin mitophagy → inhibits AEC senescence → alleviates fibrosis	(Chu et al., 2024)
AEC mitochondrial protection	Human alveolar epithelioid cells (A549)	PINK1-Parkin	ACSL1 ↑PINK1/Parkin mitophagy → ↓mitochondrial damage → ameliorates fibrosis	(Lin et al., 2024)
	human alveolar epithelioid-like cells (A549) and mouse	PINK1	ATF3 represses PINK1 transcription → ↓mitophagy → ↑mitochondrial dysfunction and fibrosis	(Bueno et al., 2018)
	Mouse alveolar type II cells and lung epithelial cells (MLE-12)	PINK1	mtOGG1 alleviates mtDNA damage → prevents PINK1 depletion → attenuates fibrosis	(Kim et al., 2020)
AEC metabolism	Human alveolar epithelioid cells (A549)	-	ACSS3 inhibits mitophagy via CPT1A deficiency → metabolic reprogramming → attenuates fibrosis	(Wang et al., 2024b)
AEC inflammation	Human alveolar epithelioid-like cells (A549 and ATCC CCL-185) and mouse	PINK1	PINK1 deficiency → mtDNA release → TLR9 activation → TGF-β secretion → fibrosis	(Bueno et al., 2019)
Fibroblast activation/differentiation	Pulmonary fibroblasts isolated from mouse and IPF lungs	Parkin	PARK2 knockdown → ↓mitophagy → activates PDGFR/PI3K/AKT → ↑myofibroblast differentiation	(Kobayashi et al., 2016)
	Normal human lung fibroblasts (NHLF) and mouse	p62/SQSTM1	TGF-β1 inhibits autophagy during myofibroblast differentiation → promotes fibrosis	(Sosulski et al., 2015)
	Lung fibroblasts (LF)	Parkin	Pirfenidone induces Parkin-mediated mitophagy → ↓mtROS and PDGFR-AKT-mTOR → ↓myofibroblast differentiation	(Kurita et al., 2017)
	Primary Lung Fibroblasts	PINK1,LC3B-II,Beclin-1 and P62	BMP4 promotes mitophagy → ↓TGF-β1-induced fibroblast-to-myofibroblast differentiation	(Guan et al., 2022)
	Isolation of primary LFs from the lungs of C57BL/6 male mice	Parkin/LC3B	Alamandine/MrgD axis → ↑Parkin/LC3B mitophagy → blocks TGF-β1-mediated fibroblast activation	(Wang W. et al., 2023)
inflammatory cells	Mouse lung (PM2.5)	Parkin, SQSTM1/p62 and LC3B	↑Parkin/p62 and ↑LC3B II/I → ↑mitophagy → ↑NLRP3 → aggravates fibrosis	(Xu et al., 2021)
	Primary AECs (PAECs) isolated from the lungs of C57BL/6J mice	PINK1-Parkin	MitoTA293 inhibits mitophagy → activates NLRP3/caspase-1/IL1β/p38 → exacerbates fibrosis	(Sakai et al., 2021)
	human monocytes (THP-1) and mouse alveolar macrophages (MH-S), mouse	LC3-II,PINK1 and Parkin	Akt1 ↑PINK1/Parkin → ↑mitophagy → ↑mtROS → ↑TGF-β1 → promotes fibrosis	(Larson-Casey et al., 2016)
	Macrophages (RAW264.7), mouse	PINK1,Parkin,p62 and LC3B	*Klebsiella* triggers macrophage mitophagy → promotes IPF progression	(Xu et al., 2025)
	IPF patients AMs	PINK1,Parkin and NRF1	↓PINK1/PARK2/NRF1 in AMs → defective mitophagy → fibrosis	(Tsitoura et al., 2019)
	primary mouse macrophages	PINK1-Parkin	miR-33 deficiency → ↑mitophagy → ↓inflammation → antifibrotic	(Ahangari et al., 2023)
	Human monocytic leukemia cells (THP-1)/HFL-1	PINK1-Parkin	OGG1 knockdown → ↑PINK1/Parkin → attenuates M2 macrophage-induced mitochondrial dysfunction	(Wu et al., 2024)
	AECs (HPAEpiC), lung fibroblasts (HLF-1) and mouse	PINK1	Thymosin β4 ↑PINK1 → ↓NLRP3/IL-1β → ↓inflammation and fibrosis	(Tian et al., 2022)

### 3.1 Mitophagy is involved in oxidative stress

Mitochondria are a major source of ROS, and oxidative stress can contribute to the progression of IPF by secreting a variety of cytokines as an important link in the initiation and development of IPF. Impaired mitophagy and aberrant redox signaling by excess ROS promote IPF progression (Ornatowski et al., 2020). Emerging evidence suggests that TET is a potent inducer of autophagy and plays a role in the treatment of inflammatory diseases (Bhagya and Chandrashekar, 2016; Song et al., 2022). TET may ameliorate cellular senescence by affecting the ROS loop of impaired mitophagy (CHU et al., 2024). Meanwhile, SIRT1, a known regulator of autophagy and senescence, was shown that cigarette smoke (CS)-induced senescence of alveolar type II epithelial cells (AT2) was associated with a decrease in autophagy mediated by SIRT1 inactivation, while the decrease in autophagy could inhibit SIRT1 activity by promoting the expression of mitoROS (Zhang Y. et al., 2021). Bueno et al. found that in IPF patients and aged mice, the activation of transcription factor 3, a key repressor of PINK1 transcription, resulted in increased mitochondrial ROS production and mitochondrial depolarization (Bueno et al., 2018). PFD, one of the FDA-approved antifibrotic drugs for the treatment of IPF, acts to reduce ROS production by promoting Parkin-mediated mitophagy (Kurita et al., 2017). PINK1/Parkin-mediated mitophagy eliminates mitochondrial ROS production and inhibits fibroblast differentiation (Kobayashi et al., 2016; Álvarez et al., 2017; Malsin et al., 2018; Lei and Kazlauskas, 2014).

However, conflicting reports also exist on mitophagy and oxidative stress during IPF. In alveolar macrophages (AM), overexpression of protein kinase B1 (Akt1) upregulates PINK1 and Parkin expression, enhances mitophagy, promotes mtROS production, and exacerbates the process of pulmonary fibrosis (Larson-Casey et al., 2016), suggesting that enhanced mitophagy instead promotes oxidative stress in alveolar macrophages. A plausible explanation is that IPF is not a static process, but an evolving disease state with increasing lung and mitochondrial damage as the disease progresses.

The above studies indicate that the relationship between mitophagy and oxidative stress in IPF is complex, and the level of mitophagy varies significantly among different cell types. In the future, we should strengthen the study of the different stages of IPF to reveal the dynamic changes of mitophagy in the course of the disease more comprehensively.

### 3.2 Mitophagy is involved in the regulation of AECs

AECs are important cells for maintaining normal alveolar epithelial homeostasis and repairing damaged epithelium (Katzen and Beers, 2020; Moss et al., 2022). Injury to AECs and aberrant repair are the main mechanisms leading to IPF. Recently, it has been found that IPF results from the inability of alveolar epithelium to regenerate properly as well as from repeated epithelial cell injury (Liang et al., 2022). When mitophagy is dysfunctional, mitochondrial transmembrane potential is lost and mitochondrial dynamics are abnormal, leading to alterations in the metabolic pathways of AECs which in turn lead to apoptosis and senescence, *etc.*, and affecting the development of IPF (Malsin et al., 2018; Moss et al., 2022).

Mitophagy is involved in apoptosis in AECs. As early as 2015, mitochondrial abnormalities and dysfunction were observed in AECs from PINK1-deficient mice with an increased tendency to apoptosis (Bueno et al., 2015). Subsequently, several research teams found that impaired mitophagy caused by insufficient PINK1 expression promotes apoptosis and drives fibrogenesis in AECs, both in lung tissues of IPF patients and in cellular animal models; whereas enhancement of PINK1 reverses this process, improves mitochondrial function and inhibits cell death (Bueno et al., 2015; Patel et al., 2015; Sun et al., 2020; Chen et al., 2025). Recent studies have further confirmed that Xiao H et al. found that interleukin-17A (IL-17A) knockout mice exhibit reduced levels of apoptosis in AECII, IL-17A promotes apoptosis in AECII cells by inhibiting PINK1-mediated mitophagy, exerting its pro-fibrotic effects (Xiao et al., 2022). FBR-2 formula (FBR-2) inhibits H_2_O_2_-induced activation of the PINK1/Parkin pathway, which in turn activates mitophagy, reduces apoptosis, and attenuates oxidative damage in AECs (Gu et al., 2022). Phosphoglycerate translocase family member 5 (PGAM5) deficiency inhibits PINK1-mediated mitophagy, reduces the cytotoxicity of bleomycin (BLM) on AECs, decreases apoptosis, and slows IPF (Ganzleben et al., 2019). Meanwhile, PINK1 deficiency promotes the clearance of damaged mitochondria and maintains mitochondrial function by up-regulating the BNIP3 and FUNDC1 pathways, alleviating alveolar epithelial damage and attenuating fibrosis (Gu et al., 2025). This also explains that the defective mitophagy induced in the presence of PGAM5 deficiency instead ameliorated pulmonary fibrosis, and a plausible explanation is that PGAM5 may attenuate IPF by upregulating other mitophagy pathways.

Mitophagy is involved in the senescence of AECs. Single-cell transcriptome analysis revealed the presence of a large number of senescent AECs in the lungs of patients with IPF (Reyfman et al., 2019), and the elimination of senescent cells inhibited pulmonary fibrosis (Schafer et al., 2017). Senescence of AECs is thought to be a key cause of a variety of age-related lung diseases including IPF (Chin et al., 2023). In animal models, senolytics treatment reduces pulmonary fibrosis (Spina et al., 2024; Zhang et al., 2022).

Mechanistically, mitophagy failure directly fuels the senescence phenotype in AECs. Persistent mitochondrial damage caused by defective mitophagy leads to sustained ROS production and mtDNA release, which activate the cGAS-STING pathway and promote the senescence-associated secretory phenotype (SASP). SASP factors, including IL-6, IL-8, and TGF-β, in turn create a pro-fibrotic microenvironment that activates adjacent fibroblasts. Moreover, impaired mitophagy compromises the mitochondrial–lysosomal axis, a hallmark of cellular senescence, leading to the accumulation of dysfunctional organelles and metabolic insufficiency. Notably, the transcription factor ATF3, which is upregulated in aged lungs, represses PINK1 transcription, creating a vicious cycle of mitophagy decline and accelerated senescence (Bueno et al., 2018). Consistent with this mechanistic framework, pharmacological activation of mitophagy has shown therapeutic promise. The data show that EF24, a derivative of curcumin, has anti-inflammatory, anti-tumor, and anti-aging properties. In an aged IPF mouse model, EF24 induced the reversal of persistent pulmonary fibrosis and promoted regression of fibrosis. In addition, EF24 showed better efficacy in the treatment of IPF compared to other FDA-approved drugs such as PFD and nintedanib (Zhang et al., 2024). EF24 inhibits the senescence of AECs through the activation of mitophagy, which alleviates pulmonary fibrosis (Zhang et al., 2024). Similarly, TET, an activator of autophagy, plays an important role in the treatment of inflammation-related diseases (Bhagya and Chandrashekar, 2016; Song et al., 2022). TET inhibits AEC senescence by promoting PINK1/Parkin-mediated mitophagy, thereby alleviating pulmonary fibrosis (CHU et al., 2024). Several other studies have further shown that insufficient mitophagy accelerates the pathogenesis of IPF in AECs (Bueno et al., 2019; Bueno et al., 2018; Lin et al., 2024; Kim et al., 2020). Thus, restoring mitophagy not only removes damaged mitochondria but also interrupts the senescence–SASP–fibrosis cascade, highlighting a dual therapeutic benefit. Targeting senescent AECs via mitophagy enhancement therefore emerges as a promising therapeutic strategy for IPF.

### 3.3 Mitophagy is involved in fibroblast activation and transformation

Pulmonary fibroblasts are an important component of the mesenchyme, are major producers of extracellular matrix, and are involved in wound healing. IPF is characterized by progressive scarring of lung tissue, driven primarily by activation of pulmonary fibroblasts into myofibroblasts and overproduction of the ECM component, and emerging research has highlighted the critical role of mitophagy in this pathological process.

Mitophagy is involved in the proliferation and differentiation of myofibroblasts. In contrast to PINK1, Parkin may play a major role in the regulation of myofibroblast differentiation. Animal models demonstrated that deficient mitophagy in fibroblast foci may be caused by knockdown of Parkin activating the PDGFR/PI3K/AKT signaling pathway, which in turn drives increased myofibroblast differentiation and proliferation (Kobayashi et al., 2016). PFD induces autophagy/activation of mitophagy by enhancing Parkin expression, and in the presence of transforming growth factor β (TGF-β), Parkin expression is partially involved in the inhibition of myofibroblast division (Kurita et al., 2017). Bone morphogenetic protein 4 (BMP4) enhanced the autophagic activity of damaged mitochondria in lung fibroblasts, which in turn slowed TGF-β1-driven fibroblast differentiation into myofibroblasts (Guan et al., 2022).

Mitophagy is involved in fibroblast activation. IPF-based pathogenesis involves multiple cell types and multiple pathophysiologic processes, which makes it difficult for a single therapeutic target to be effective. Interestingly, a recent study proposed a previously unknown mechanism whereby the Alamandine/MrgD axis prevents TGF-β1-mediated fibroblast activation by regulating aerobic glycolysis and mitophagy. The Alamandine/MrgD axis inhibited TGF-β1-induced fibroblast activation, thus exerting an antifibrotic effect (Wang W. et al., 2023). However, the relationship between Alamandine expression and glycolysis/mitophagy in IPF patients has not yet been evaluated in depth and requires further validation.

Collectively, the role of mitophagy in fibroblast activation extends beyond ROS management to encompass metabolic reprogramming. In normal lung fibroblasts, oxidative phosphorylation (OXPHOS) dominates energy production; however, when mitophagy is defective, mitochondrial dysfunction forces a metabolic shift toward aerobic glycolysis (the Warburg effect) (Zhu et al., 2026). This glycolytic switch stabilizes hypoxia-inducible factor-1α (HIF-1α), which directly upregulates pro-fibrotic genes such as TGF-β1, α-SMA, and collagen I. Moreover, the accumulation of mitochondrial ROS sustains the activation of PDGFR/PI3K/AKT and TGF-β/Smad3 signaling pathways, creating a positive feedback loop that drives myofibroblast differentiation and excessive extracellular matrix deposition (Li et al., 2020). Intriguingly, the Alamandine/MrgD axis simultaneously inhibits this glycolytic switch and promotes mitophagy, suggesting that concurrent metabolic and organellar regulation may be key to controlling fibroblast activation (Zhang et al., 2025). Therefore, therapeutic strategies that both restore mitophagy and normalize metabolic flux could more effectively halt fibrosis progression than targeting either process alone.

### 3.4 Involvement of mitophagy in the inflammatory response

In IPF, inflammatory cells initiate inflammatory responses, secrete inflammatory factors, and remodel the microenvironment during early pathological processes. Mitophagy, an important intracellular metabolic regulatory mechanism, may interact with the inflammatory response to influence the course of IPF.

First, mitophagy was involved in the inflammatory response in IPF by regulating the inflammatory signaling pathway. The expression of autophagy-related proteins was upregulated and NLRP3 levels were significantly upregulated in the lung tissues of PM2.5-exposed mice, suggesting that enhanced mitophagy may activate the inflammatory response (Xu et al., 2021). In addition, in the study of Rimessi et al. (Mizumura et al., 2014), Parkin expression was reduced during *Pseudomonas aeruginosa* infection, the ratio of LC3BII/LC3B I was decreased, and the expression of NLRP3, IL-1β, and IL-18 was upregulated, and the inflammatory response was activated, aggravating lung fibrosis, confirming that insufficient mitophagy activates the inflammatory response and aggravates lung fibrosis. MitoTA293, a mitochondria-OH-targeted antioxidant, inhibited the induction of mitophagy and enhanced the activation of the NLRP3 inflammatory vesicle/caspase-1/IL1β/IL1R/p-p38 MAPK/p16 and p21 pathways, while exacerbating cellular senescence, inflammation, and fibrosis (Sakai et al., 2021). At the same time, reduced mitophagy also leads to the extracellular release of mitochondrial damage-associated molecular patterns (mtDAMPs), such as mtDNA, which induces pro-inflammatory cell signaling via Toll-like receptor 9 (TLR9) (Bueno et al., 2019).

Mitophagy is involved in the inflammatory response in IPF by reducing ROS production. For example, lipopolysaccharide (LPS) inhibits mitophagy in HPAEpiC cells and promotes inflammation through oxidative stress, and defective mitophagy leads to impaired ROS clearance and accumulation and activation of NLRP3 inflammatory vesicles (Tian et al., 2022). Meanwhile, the role of redox imbalance in IPF is discussed in this article, specifically how mitochondrial dysfunction promotes inflammatory responses and fibrosis by increasing ROS production, suggesting that activation of mitophagy reduces ROS production, thereby attenuating inflammation and fibrosis (Veith et al., 2019). Akt1 is known for mediating mitochondrial H_2_O_2_ production, and Akt1 overexpression in AM mediated inhibition of mitochondrial ROS on mitophagy eliminates TGF-β expression and fibroblast differentiation (Larson-Casey et al., 2016).

Mitophagy influencing immune cell polarization is involved in the inflammatory response in idiopathic pulmonary fibrosis. Mitophagy in fibroblasts may affect M2 polarization and create a feedback loop between M2 macrophages and fibroblasts, although the effect may be small (Wu et al., 2024). miR-33 deletion in AM augmented mitochondrial homeostasis and autophagy, while alleviating BLM-induced inflammation. AM extracted from WT mice treated with PNA-33 (miR-33 inhibitor) differentiated in the direction of M1 (using INF-γ + LPS) or M2 (using IL-13), respectively (Ahangari et al., 2023).

The inflammatory response is not only a trigger for fibrosis in IPF, but also continues to drive disease progression through interactions with other mechanisms (e.g., oxidative stress, mitochondrial dysfunction, and immune cell abnormalities). Although anti-inflammatory therapy is effective in some patients, its application needs to be combined with disease stage and individual heterogeneity. Future studies should focus on the dynamic regulation of inflammatory networks and multi-target combined intervention strategies.

Overall, mitophagy plays a key role in IPF by regulating oxidative stress, apoptosis, senescence, inflammatory response, and fibroblast activation and differentiation. Further exploration of the regulatory mechanisms and potential therapeutic targets of mitophagy may provide new strategies for the treatment of IPF.

### 3.5 Conflicting evidence and contextual determinants of mitophagy in IPF

Despite substantial evidence supporting the protective role of mitophagy in IPF, contradictory findings exist. For instance, while PINK1 deficiency in AECs promotes fibrosis and apoptosis (Bueno et al., 2015; Patel et al., 2015), other studies demonstrate that PINK1 ablation alleviates BLM-induced fibrosis via compensatory upregulation of BNIP3 and FUNDC1 (Gu et al., 2025). This discrepancy suggests that the dependency on distinct mitophagy pathways varies across experimental contexts and cell types. Similarly, although enhanced mitophagy generally limits oxidative injury in AECs, Akt1-mediated upregulation of mitophagy in alveolar macrophages promotes mtROS production and exacerbates fibrosis (Larson-Casey et al., 2016). These opposing outcomes underscore that the pathological impact of mitophagy is highly cell-type-specific and context-dependent. Future studies should integrate single-cell resolution approaches to dissect the spatiotemporal dynamics of mitophagy in the heterogeneous IPF lung microenvironment.

## 4 The potential of modulating mitochondrial autophagy for the treatment of IPF

IPF research has continued for many years, yet clinical interventions remain largely palliative, and multiple promising treatments have failed in clinical trials, possibly due to the complex mechanisms underlying the disease (Pokharel et al., 2024). Targeting mitophagy represents a promising strategy, as emerging evidence highlights its critical role in IPF pathogenesis (Li S. et al., 2022).

Lung cells from IPF patients exhibit reduced levels of PINK1 and Parkin, indicating impaired mitophagy (Araya et al., 2013; Sun et al., 2020). PINK1-deficient mice are more susceptible to pulmonary fibrosis and show significant mitochondrial dysfunction (Sun et al., 2020). In the context of deficient mitophagy, PFD induces Parkin-mediated mitophagy and suppresses lung fibrosis progression (Kurita et al., 2017). These findings suggest that activating the mitophagy pathway may confer therapeutic benefits. Current potential therapeutic strategies are summarized below and in Table 3.

**TABLE 3:** Drugs with potential for IPF treatment.

Category	Drug	Experimental model	Mitophagy-related targets	Functionality	References
Biological drugs	Metformin	IPF lung tissue, fibroblasts, rat, THP1, HBEC	Beclin1, LC3B, P62, AMPK-mTOR	Activates AMPK↑→↓mTOR-dependent S6 phosphorylation→ autophagy↑ →alleviate pulmonary fibrosis	(Rangarajan et al., 2018; Li et al., 2021)
	Rapamycin	Human lung fibroblasts (MRC-5), mouse, AECs	Beclin1, LC3, P62, LC3II/I	Inhibits mTOR overactivation↓,autophagy↑→ exerts antifibrotic	(Wang et al., 2018; Gui et al., 2015)
	KTP	Dopamine neurons	PINK1-Parkin	Enhances PINK1/Parkin activity→ reduces apoptosis markers↓	(Hertz et al., 2013)
	MitoQ (mitochondria-targeted antioxidant)	A549 cells, rat	PINK1-Parkin	mitophagy↑→attenuates fibrosis	(Lin et al., 2024; Ning et al., 2021)
	USP30 inhibitors	hTERT-RPE1-YFP-Parkin cells	TOM20-Parkin	USP30 knockdown↓→ promotes mitophagy ↑and substrate ubiquitination	(Liang et al., 2015; Cunningham et al., 2015; Adnot et al., 2019)
	ACSL1	A549, IPF patients, rat	PINK1-Parkin	PINK1-Parkin Overexpression →mitochondrial damage↓,restores mitophagy→ ameliorates fibrosis	(Lin et al., 2024)
	OGG1 inhibitors	HFL-1 fibroblasts, mouse	PINK1-Parkin	OGG1 Knockdown →PINK1/Parkin↑, →attenuates M2 macrophage-induced mitochondrial dysfunction	(Wu et al., 2024; Tanner et al., 2023)
Natural products	EF24 (curcumin analog)	A549, ATII cells	LC3BII,P62, VADC1	Activates PTEN→AKT/mTOR/NF-κB↓→induces mitophagy→ inhibits AEC senescence	(Zhang et al., 2024)
	TET	MLE-12 cells	PINK1-Parkin	Promotes mitophagy→inhibits AECsenescence→alleviates fibrosis	(Chu et al., 2024)
	FBR-2	A549 cells	PINK1,Parkin, LC3B-II	Activates mitophagy, balances mitophagy/apoptosis inhibits oxidative stress-induced injury	(Gu et al., 2022)
	Berberine	Rat	Beclin-1,LC3-II, PI3K/Akt-mTOR	Upregulates PTEN↑→promotes autophagy→ attenuates BLM-induced fibrosis	(Chitra et al., 2015)
	Naringin	Mouse	PINK1, ATF3/PINK1 axis	viaATF3/PINK1→Promotes mitophagy↑→reduces ER stress and apoptosis	(Wei et al., 2023)
	Resveratrol (RES)	Various disease models	PINK1-Parkin, AMPK, SIRT1, SIRT3	Activates mitophagy→reduces ROS↓→maintains mitochondrial integrity	(Liu et al., 2025)
Mitochondria/Cell &Gene therapy	Mito-MEN	A549, mouse	Parkin	Promotes clearance of dysfunctional mitochondria, repairs mitochondrial function	(Wang L. et al., 2024)
	Mitochondrial transfer	Mouse	TOMM5, TOMM6	Restores mitochondrial homeostasis, reactivates suppressed mitophagy	(Huang et al., 2023)
	LRMSCs	IPF patients	PINK1	IPF-derived LRMSCs show impaired mitochondrial respiration and autophagic response	(Mercader-Barceló et al., 2023)
	BMP4 gene therapy	Mouse, primary lung fibroblasts	PINK1	Inhibits TGF-β1↓→induced fibroblast activation→ promotes mitophagy↑→suppresses fibrosis	(Guan et al., 2022)

Abbreviations: FBR-2, Feibi Recipe No. 2; OGG1, 8-oxoguanine DNA, glycosylase; KTP, kinetin triphosphate (KTP); ACSL1, long-chain fatty acyl-CoA, synthetase 1; Mito-MEN, mitophagy-enhancing nanoparticles; LRMSCs, lung-resident mesenchymal stem cells (MSCs); BMP4.

### 4.1 Biological drugs

Metformin, a first-line therapy for type 2 diabetes, activates autophagy via the AMPK-mTOR signaling pathway and attenuates lung fibrosis in BLM- and silica-induced models (Rangarajan et al., 2018; Li et al., 2021). A national cohort study further demonstrated that metformin use was associated with a 54% reduction in all-cause mortality among IPF patients with type 2 diabetes (Teague et al., 2022). Rapamycin, an mTOR inhibitor, has shown antifibrotic effects by restoring autophagy in IPF cellular and animal models (Wang et al., 2018; Gui et al., 2015; Hertz et al., 2013).

Kinetin triphosphate, a synthetic bioactive molecule, upregulates PINK1/Parkin activity and downregulates apoptosis-related markers, suggesting therapeutic potential against fibrosis (Hertz et al., 2013). The mitochondria-targeted antioxidant MitoQ has been reported to enhance PINK1/Parkin-mediated mitophagy and attenuate pulmonary fibrosis in preclinical models (Lin et al., 2024); however, other studies suggest it may inhibit mitophagy in certain contexts, indicating context-dependent effects (Ning et al., 2021).

Ubiquitin-specific protease 30 (USP30), a mitochondria-localized deubiquitinating enzyme, negatively regulates PINK1/Parkin-mediated mitophagy. USP30 knockdown or pharmacological inhibition promotes mitophagy and has shown efficacy comparable to PFD in pulmonary fibrosis models (Liang et al., 2015; Cunningham et al., 2015; Adnot et al., 2019; Roque et al., 2020; Bingol et al., 2014; Nakamura and Hirose, 2008; Yue et al., 2014; Kluge et al., 2018). Additionally, long-chain acyl-CoA synthetase 1 (ACSL1) is downregulated in IPF lung tissue, and its overexpression reduces mitochondrial damage and activates PINK1/Parkin-mediated mitophagy (Lin et al., 2024). Similarly, inhibition of 8-oxoguanine DNA glycosylase 1 (OGG1) promotes PINK1/Parkin expression and attenuates M2 macrophage-induced fibroblast dysfunction (Wu et al., 2024; Tanner et al., 2023).

### 4.2 Natural products

Natural products offer advantages such as multi-target effects, lower toxicity, and reduced drug dependence compared to single-target agents. EF24, a curcumin analog, inhibits AEC senescence by activating PTEN and inducing mitophagy (Zhang et al., 2024). TET promotes PINK1/Parkin-mediated mitophagy and alleviates pulmonary fibrosis by suppressing AEC senescence and fibroblast activation (Chu et al., 2024). The traditional Chinese medicine formula FBR-2 regulates the mitophagy/apoptosis balance and protects A549 cells from oxidative stress-induced injury (Gu et al., 2022).

Berberine, an isoquinoline alkaloid, enhances autophagy and attenuates BLM-induced fibrosis by inhibiting the PI3K/Akt/mTOR pathway (Chitra et al., 2015). Naringin alleviates pulmonary fibrosis by activating the ATF3/PINK1 signaling axis, thereby reducing ER stress and maintaining mitochondrial homeostasis (Wei et al., 2023). Resveratrol (RES), a polyphenolic compound, activates mitophagy through AMPK, SIRT1, and SIRT3, reducing ROS production and preserving mitochondrial integrity (Liu et al., 2025).

The multi-target, multi-pathway nature of natural products underscores their therapeutic potential; however, further mechanistic studies are needed to fully elucidate their roles in mitophagy regulation and IPF treatment (Shi et al., 2024).

### 4.3 Mitochondria/cell and gene therapy

Recent advances in mitochondrial and gene therapy have opened new avenues for IPF treatment. Engineered mitochondria-enhancing nanoparticles (Mito-MEN) promote the synchronized clearance of dysfunctional mitochondria and have shown efficacy in alleviating pulmonary fibrosis (Wang L. et al., 2024). Mesenchymal stem cell (MSC)-mediated mitochondrial transfer can restore mitochondrial function in damaged lung cells by reactivating suppressed mitophagy (Huang et al., 2023; Li et al., 2019; Ahmad et al., 2014). Lung-resident MSCs (LRMSCs) isolated from IPF patients exhibit impaired mitochondrial respiration and autophagic responses, suggesting that restoring mitophagy in MSCs may represent a novel therapeutic strategy (Mercader-Barceló et al., 2023).

Building on the promise of cell-based approaches, MSC-derived exosomes (MSC-Exo) have recently emerged as a cell-free alternative that bypasses the safety and logistical concerns of live cell infusion (Sandonà et al., 2021). In the context of pulmonary fibrosis, MSC-Exo carry bioactive cargoes, including microRNAs (e.g., miR-17–92 cluster, miR-30b) and even intact mitochondrial components, which can be transferred to injured alveolar epithelial cells. Upon uptake, these exosomes restore mitochondrial membrane potential, re-establish PINK1/Parkin-mediated mitophagy flux, and reduce apoptosis in bleomycin-injured lungs (Harrell et al., 2024). Mechanistically, MSC-Exo appear to upregulate Parkin expression via AKT/FOXO3a signaling, thereby enhancing the clearance of dysfunctional mitochondria (Wei et al., 2025). Importantly, systemic administration of MSC-Exo in murine models not only attenuated fibrosis but also reduced senescence markers, suggesting that exosome-based mitophagy restoration may offer a dual advantage of epithelial protection and anti-aging effects in IPF (Wang J. et al., 2024). Clinical translation, however, requires standardized isolation protocols and rigorous efficacy testing in IPF patient-derived models.

Beyond cell- and exosome-based strategies, direct gene therapy has also shown preclinical promise. Bone morphogenetic protein 4 (BMP4) gene therapy, delivered via adeno-associated virus 9 (AAV9), has been shown to inhibit TGF-β1-induced fibroblast activation, promote mitophagy, and attenuate BLM-induced lung fibrosis in mice (Guan et al., 2022). Although no gene therapy is currently approved for IPF, strategies targeting key pathogenic genes (e.g., TGF-β1, NOX4, SMAD3) have shown promising results in preclinical models (D'alessandro-Gabazza et al., 2012; Hecker et al., 2009; Dong et al., 2012; Lagares et al., 2012).

### 4.4 Combination therapeutic strategies based on mitophagy modulation

Given the multifactorial nature of IPF—involving epithelial injury, fibroblast activation, inflammation, and metabolic reprogramming—targeting mitophagy alone may be insufficient. Combination strategies that leverage synergistic mechanisms could improve therapeutic outcomes.

#### 4.4.1 Combination with existing antifibrotic agents

PFD has been shown to induce Parkin-mediated mitophagy (Kurita et al., 2017); combining it with other mitophagy enhancers (e.g., MitoQ or USP30 inhibitors) may yield additive or synergistic effects. Preclinical data suggest that co-administration of PFD and MitoQ more effectively reduces mitochondrial oxidative stress and suppresses fibroblast activation. Nintedanib, a tyrosine kinase inhibitor, also holds promise in combination with mitophagy modulators, potentially achieving multi-dimensional intervention by simultaneously targeting fibroblast activation and mitochondrial dysfunction.

#### 4.4.2 Combination with anti-inflammatory/senolytic agents

Given the close association between defective mitophagy, cellular senescence, and inflammasome activation, combining mitophagy modulators with senolytics (e.g., dasatinib plus quercetin) or NLRP3 inflammasome inhibitors may enhance efficacy. Quercetin itself has mitophagy-modulating properties, and such combinations could concurrently clear senescent cells and restore mitochondrial quality control.

#### 4.4.3 Combination with cell-based and gene therapies

MSC-mediated mitochondrial transfer can restore mitochondrial function in injured cells, and mitophagy modulators may improve the integration and utilization of transferred mitochondria. Furthermore, gene therapy (e.g., AAV9-mediated BMP4 delivery) combined with pharmacological agents could offer both long-term disease modification and rapid symptom control.

#### 4.4.4 Clinical development considerations for combination therapy

Translating combination strategies into clinical practice requires careful evaluation of drug–drug interactions, dosing schedules, and patient stratification. Future trial designs should prioritize combinations with complementary mechanisms and use biomarkers (e.g., circulating mtDNA, PINK1/Parkin expression levels) to identify likely responders. The clinical development status of representative mitophagy modulators is summarized in Table 4.

**TABLE 4:** Clinical development status of representative mitophagy modulators.

Agent/Intervention	Mechanism of action	Clinical stage	Route	Pharmacokinetic/safety profile	Main limitations
Metformin	AMPK activation, induces mitophagy	Preclinical/retrospective cohort	Oral	Good safety profile; limited lung exposure	Non-specific; high doses required
Rapamycin	mTOR inhibition	Preclinical	Oral/inhaled	Immunosuppressive side effects; narrow therapeutic window	Systemic toxicity limits long-term use
MitoQ	Reduces mtROS; indirectly modulates mitophagy	Phase I/II (for other indications)	Oral	Lung enrichment; short half-life	Not yet in trials for IPF
USP30 inhibitors	Enhance PINK1/Parkin-mediated mitophagy	Preclinical	Oral	High target specificity; some candidates in early development	Still in drug discovery phase
MSCs	Mitochondrial transfer; modulate autophagy	Phase I/II	Intravenous	Generally safe; mild fever common	Variable efficacy; unclear mechanism
AAV9-BMP4 gene therapy	Promotes mitophagy; inhibits fibroblast activation	Preclinical	Intratracheal	Long-lasting expression (≥6 weeks); local delivery reduces systemic risk	Clinical translation requires safety and immunogenicity validation

Due to the short overall research time, the study of mitochondrial autophagy regulators is still in the preliminary stage, and most of the data come from preclinical studies. Combining animal models of disease with an *in vivo* mitochondrial autophagy imaging system will help to reveal the etiology and progression of the disease and facilitate translational research.

## 5 Challenges and prospects

Despite growing evidence supporting the critical role of mitophagy in the pathogenesis of IPF, several challenges remain in translating these mechanistic insights into clinical applications. Addressing these challenges will be essential for developing effective mitophagy-targeted therapies.

### 5.1 Challenges

#### 5.1.1 Limitations of current experimental models

The most widely used animal model of IPF is the BLM-induced pulmonary fibrosis model. Although it recapitulates certain aspects of fibrosis, this model has significant limitations. BLM primarily causes acute epithelial injury followed by reversible fibrosis, whereas human IPF is a chronic, progressive, and irreversible disease characterized by heterogeneous fibroblastic foci and a distinctive aging-associated pathogenesis. Moreover, the BLM model does not fully reproduce the complex genetic, epigenetic, and environmental interactions underlying human IPF. These discrepancies may explain why many promising drug candidates have failed in clinical trials despite showing efficacy in preclinical models.

#### 5.1.2 Heterogeneity and dynamics of mitophagy

Mitophagy levels vary substantially across different cell types (e.g., AEC, fibroblasts, macrophages) and disease stages. Most current studies assess mitophagy at a single time point using static markers (e.g., LC3B, PINK1, Parkin), which fails to capture the dynamic nature of this process. Additionally, conflicting reports exist regarding whether enhanced mitophagy is protective or detrimental in IPF, suggesting that the role of mitophagy may be context-dependent. For instance, while PINK1 deficiency in AECs promotes fibrosis, enhanced mitophagy in macrophages may exacerbate inflammatory responses. Such cell-type- and stage-specific effects complicate the development of globally targeted interventions.

#### 5.1.3 Lack of reliable biomarkers and non-invasive monitoring tools

Currently, there are no validated non-invasive biomarkers to assess mitophagy flux in patients. Most evidence is derived from tissue samples or cultured cells, limiting the ability to monitor disease progression or therapeutic response in real time. The development of imaging tools or circulating biomarkers (e.g., mtDNA, mitophagy-related exosomes) that reflect mitophagy activity in the lung remains an unmet need.

#### 5.1.4 Clinical translation hurdles

Although several drugs and natural products have been shown to modulate mitophagy in preclinical models, none have been specifically approved for IPF based on mitophagy-targeting mechanisms. Challenges include poor bioavailability, off-target effects, and the lack of disease-specific delivery systems. Furthermore, the two FDA-approved drugs, PFD and nintedanib, only slow disease progression and do not reverse established fibrosis, underscoring the need for more effective and mechanism-based therapies.

### 5.2 Prospects

#### 5.2.1 Identification of novel mitophagy regulators

Beyond the well-studied PINK1/Parkin pathway, recent studies have identified additional regulators such as USP30, FUNDC1, BNIP3, and PGAM5 that may serve as more specific therapeutic targets. For example, USP30 inhibitors have shown promise in enhancing mitophagy and reducing fibrosis in preclinical models, with some candidates already entering early-stage drug development. Targeting such upstream or alternative regulators may offer greater specificity and fewer off-target effects compared to broad autophagy modulators like rapamycin or metformin.

#### 5.2.2 Development of cell-type-specific and stage-specific interventions

Given the divergent roles of mitophagy across different lung cell populations, future therapeutic strategies should aim for cell-type-specific modulation. Advances in nanoparticle-based delivery systems, such as lung-targeting liposomes or exosomes, may enable selective delivery of mitophagy modulators to AECs or fibroblasts. Additionally, stage-specific interventions—such as promoting mitophagy in early disease to prevent epithelial injury, or inhibiting excessive mitophagy in late-stage fibrotic foci—could improve therapeutic outcomes.

#### 5.2.3 Integration of non-invasive monitoring tools

The application of novel imaging techniques, such as positron emission tomography (PET) tracers targeting mitochondrial membrane potential or mitophagy-associated proteins, could enable real-time assessment of mitophagy activity in the lungs. Additionally, liquid biopsy approaches—including quantification of circulating mtDNA, mitochondrial-derived vesicles, or exosomal mitophagy-related proteins—hold promise as minimally invasive biomarkers for patient stratification and treatment monitoring.

#### 5.2.4 Multi-omics and precision medicine approaches

High-throughput technologies, including transcriptomics, proteomics, and metabolomics, offer opportunities to characterize mitophagy-related molecular signatures in IPF patients. Integration of these data with clinical parameters could facilitate the identification of patient subgroups that are more likely to benefit from mitophagy-targeting therapies. Such precision medicine strategies may improve clinical trial success rates by enriching for responsive populations.

#### 5.2.5 Combination therapies

Given the multifactorial nature of IPF, it is unlikely that targeting mitophagy alone will suffice. Future therapeutic regimens may involve combination strategies that simultaneously address mitochondrial dysfunction, oxidative stress, inflammation, and fibroblast activation. For instance, combining a mitophagy enhancer with an existing antifibrotic agent could yield synergistic effects and potentially reverse established fibrosis rather than merely slowing progression.

### 5.3 Concluding remarks of the section

In summary, while the therapeutic potential of targeting mitophagy in IPF is substantial, significant challenges related to model limitations, mechanistic heterogeneity, and clinical translation must be addressed. Future research should focus on developing cell-type-specific interventions, identifying reliable biomarkers, and leveraging multi-omics approaches to enable precision medicine. With continued advances in drug delivery systems and non-invasive monitoring tools, mitophagy-targeted strategies may ultimately become a viable component of the IPF therapeutic armamentarium.

## 6 Summary

In conclusion, compelling evidence supports a multifaceted role of mitophagy in IPF pathogenesis, encompassing oxidative stress regulation, epithelial homeostasis, fibroblast activation, and inflammatory modulation. However, the translation of these mechanistic insights into clinical practice faces substantial hurdles, including model limitations, cell-type-specific effects, and the absence of reliable biomarkers.

Currently, there is still a gap in translating mitophagy mechanism studies into effective targeted drugs; therefore, in order to effectively explore the therapeutic value of mitophagy, it is still necessary to strengthen basic biological studies and improve related drug strategies. Looking forward, the convergence of precision medicine, advanced drug delivery systems, and combinatorial therapeutic strategies holds promise for transforming mitophagy-targeted interventions from bench to bedside.
